# Design & automation of a small-scale towing tank for flow visualization

**DOI:** 10.1016/j.ohx.2024.e00585

**Published:** 2024-09-13

**Authors:** Jeremiah Takyi, Heather R. Beem

**Affiliations:** Department of Engineering, Ashesi University, 1 University Ave, Berekuso, Ghana

**Keywords:** Towing tank, Fluid mechanics, Computer Numerical Control (CNC), Flow visualization, Automation, Arduino

## Abstract

Although the towing tank is a standard piece of equipment used to investigate fluid phenomena, it primarily exists as custom-built hardware that takes up a significant footprint. The size, cost, and custom-built nature have heretofore inhibited the production of this equipment in the authors’ context, an African university. This paper presents a small-scale (1000 mm x 200 mm x 200 mm), low-cost (<$1,000) towing tank made using readily available components and basic digital fabrication tools. Other universities on the continent and beyond can hence create this foundational platform for fluid mechanics-related teaching and research. Leveraging an Arduino microcontroller loaded with the GRBL firmware, G-code is sent from the computer to stepper motors to execute movements in two axes. This allows for automation capabilities, controlled towing speeds, and consistent experimental conditions. Validation tests revealed motion accuracy within 1 %. A glitter-based flow visualization approach to measuring surface phenomena is demonstrated here. Experiments conducted successfully visualized relevant flow characteristics generated by bluff bodies being towed in the tank. As the Reynolds number increased within the operating range, wider wakes and larger, more distinct vortices were generated, as expected. This platform can be replicated widely in institutions that may otherwise forego experimentation in fluid mechanics.

## Graphical abstract

**Figure:**
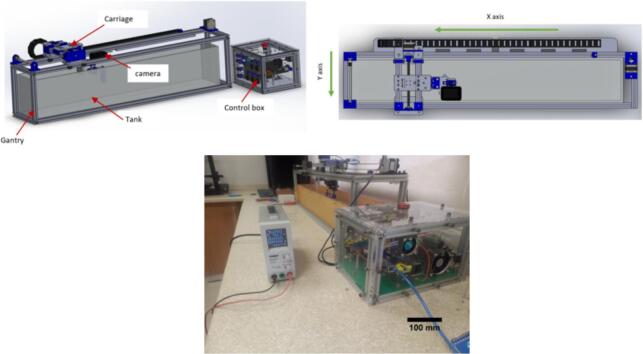

Specifications table

**Table:** 

Hardware name	Mini towing tank
Subject area	Educational tools and open source alternatives to existing infrastructure
Hardware type	• Imaging tools • Measuring physical properties and in-lab sensors • Mechanical engineering and materials science
Closest commercial analog	No commercial analog is available. These systems are mostly custom-built for specific universities or research labs.
Open source license	CC BY 4.0
Cost of hardware	GHC 9584 ($613)
Source file repository	https://doi.org/10.17632/k5y3ffzn3b.1

## 1 Hardware in context

Many universities and research labs that engage in fluid mechanics teaching or research utilize wind tunnels and/or towing tanks as their foundational experimental platforms. These have been implemented at various institutions such as [1], [2]. These facilities are used for conducting aerodynamic and hydrodynamic experimentation, analyzing fluid flow phenomena, observing the behavior of fluids around bodies and more. A standard wind tunnel is a facility in which airflow is generated around a stationary object, such as an aircraft wing or model vehicle, enabling the measurement of drag, lift, and other aerodynamic forces. A standard towing tank is one in which test objects are towed through an otherwise stationary tank of water. Due to the relative velocity theory, a specimen fastened to the carriage and towed at preset speeds in an otherwise motionless fluid is equivalent to a stationary object experiencing flow over it at the same velocity as the towing speed. Hence the standard towing tank setup enables a controlled investigation of flow behavior around various objects such as ship hulls, submarines, and marine robots, as well as measurement of respective forces they experience [3]. The two facilities can, to an extent, be used to achieve similar outcomes. Flow visualization capabilities can be integrated into any of these facilities due to the accurate velocity and acceleration profiles they provide.

A diverse set of applications have benefited from experimental studies in a towing tank facility. For example, experiments were conducted on a model Mars robot to study the fluid interference caused by a mast holding one of its mounted cameras [4]. Dye was introduced to the flow as the model was towed at a specific velocity within the tank, enabling visualization of the flow features around the specimen. The capabilities of autonomous underwater vehicle (AUV)s have been tested in these facilities, such as determining their “visual recognition control” [5]. The oil and gas industry has been able to re-design underwater piping systems and offshore structures that were adversely affected by vortex-induced vibrations (VIV). Different model pipes were tested by towing them at specific speeds and analyzing the flow patterns around them to ascertain the severity of the VIV that developed [6]. These few examples highlight the extensive use of towing tanks in different institutions and sectors.

Towing tanks are particularly uncommon in many universities in the global south, including the one where the authors are located. This is due to various factors, including the high cost of their construction and upkeep, the size of the complete setup, the logistical difficulties involved in transporting the specialized equipment, and the need for available expertise to run and maintain such facilities. Documentation exists for a few towing tanks that have been developed in lower to middle-income countries, such as [7]. Many institutions in these contexts, however, revert to “virtual” towing tanks, relying on computational approaches to predict the expected performance of the test objects [8]. Once again, high cost tends to be limiting factor as well as the space required, since a typical towing tank can span several meters, posing a challenge for such institutions who may usually have limited space for a relatively large student population.

Although the need for a small-scale towing tank that addresses the issues highlighted earlier is evident, few efforts at the small-scale have been documented. Even a tank on the order of tens of meters long would traditionally be called “small”. Nonetheless tanks one order of magnitude smaller have been used, especially in university settings. One example is a tank used at MIT for experimental fluid [9]. The 2.4-meter-long tank confined to a space envelope of less than 5 m cubed, is being used extensively for various experiments. This demonstrates the feasibility of utilizing small-scale tanks for research in fluid dynamics and still highlights a gap in creating benchtop-sized platforms.

In this work, we demonstrate a low-cost, small-scale, Computerized Numerical Control (CNC)-based towing tank designed for fluid mechanics experimentation. This hardware serves as a versatile platform for conducting diverse teaching and research endeavors within fluid mechanics. The mechanical structure primarily consists of plexiglass, 2020 aluminum extrudes, and 3D-printed parts. The electronics are centered around an Arduino microcontroller and basic electronic components such as optocouplers and DC-DC buck converters. The setup offers a scalable and cost-effective alternative to expensive proprietary systems, fostering broader access to fluid flow experimentation, particularly in the global south.

## 2 Hardware description

The small-scale (mini) towing tank, which can be seen in the figure below, is majorly composed of the tank, camera, gantry, carriage, and control box. The tank is fabricated from plexiglass and filled with water. The control box houses all the vital electronics for running the system. The carriage is programmed to move across the gantry at a user-defined speed. A test object should be mounted to the carriage such that it hangs vertically in the water. Two stepper motors provide motion in the X (in-line) and Y (cross-flow) axes, controlled by an Arduino UNO microcontroller running GRBL software. The system is operated using either open-source G-code software like Universal G-code Sender (UGS) or a custom software programmed in MATLAB. Finally, there is a mounting point for flow visualization experiments using a camera.

Compared to pre-existing methods, this hardware offers several advantages:

1. Its cost is significantly lower than proprietary towing tanks, making it more accessible to institutions with limited material budgets. The use of off-the-shelf components and open-source software contributes to cost reduction.
2. Its modular design allows for easy detachment and mobility, enabling researchers to relocate or transport the equipment conveniently.
3. The customization prospect of the hardware encourages further exploration and development in specific areas of interest, as local students/researchers can modify and adapt the setup for different experiments or designs.
4. Users requiring larger-scale experiments can easily modify the design by using longer aluminum extrusions and larger tanks.

The key limitations associated with utilizing a smaller-scale tank, such as the one described here, are in the smaller size of test specimen that can be used and the lower velocity range that can be employed. This therefore limits the operating Reynolds number regime to relatively smaller values. In order to reduce potential wall effects, the authors recommend that the width of the test specimen not exceed one-fifth the width of the tank, which in this system equals 32 mm. In order to ensure that the test specimen reaches steady state conditions for a sufficient portion of the test run, the velocity range of this system should not exceed 100 mm/s. Hence this system is limited to operating at a maximum Reynolds number of order of magnitude 10^3. The dimensions of the towing tank were predominantly dictated by the availability of 1-meter aluminum extrusions, which are cost-effective and readily accessible in the authors’ geographical location. This choice was intended to simplify the construction process, and thereby making it more feasible for users or institutions with limited resources.

This mini towing tank provides a cost-effective, customizable, and mobile solution for fluid mechanics experiments. Its affordability, ease of use, and adaptability make it an attractive alternative to existing systems, providing a feasible platform for local students and researchers to engage with fluid mechanics experimentally.

## 3 Design files summary

### 3.1 3D printed parts

All the component files for the mini towing tank were designed using SolidWorks 2022. The files for the laser-cut parts were saved in the Drawing Exchange Format (DEF), while the sections requiring 3D printing were saved in the Surface Tessellation Language (STL) format (DXF). The 3D printing was performed using a Creality Ender 3 Pro 3D printer equipped with a 0.4 mm nozzle and Polylactic Acid (PLA) filament. A 40 % infill density was consistently used for each 3D print, ensuring a balance between structural strength and material efficiency.

### 3.2 Laser cut partsThe parameters the laser cutter was set to cut or engrave are highlighted below

Laser cutter brand: Boss Laser.

Laser wattage: 70 W.

Laser cutting parameter for 5 mm plexiglass: 80 % power, 10 mm/s speed.

Laser cutting parameter for 10 mm plexiglass: 80 % power, 5 mm/s speed.

Engraving parameter: 25 % power, 350 mm/s speed.

Interval/scan gap: 0.065 mm.

### 3.3 Electronics

**Fig. 1:**
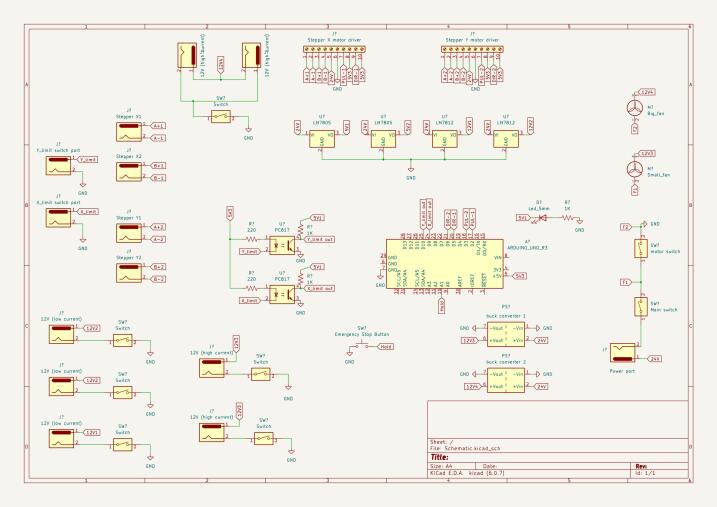
Electronic schematic diagram showing electronic component connections.

Fig. 1 below presents the schematic diagram for the complete setup of the mini-towing tank. The system has a total power requirement of 72 W, with a voltage of 24 V and a current of 3 A. The electronic circuit comprises various components, including DM556 stepper motor drivers, an Arduino UNO, an emergency stop button, optocouplers, fans, and limit switches.

It is worth noting that the use of barrel jack is optional. One might choose to directly connect the cables from the stepper motors and limit switch to the control box, bypassing the need for a barrel jack interface. While this approach is simpler, it compromises the modularity of the overall system. Additionally, the section highlighted in blue on the schematic diagram represents optional connections (extra 12 V ports), which allow users to incorporate other functionalities according to their specific design requirements or preferences. This adaptability enables customization and expansion of the mini towing tank system to suit different research or experimentation needs.

### 3.4 Software

The mini towing tank is controlled using Universal G-code Sender, an open-source software. The Arduino UNO microcontroller is loaded with GRBL firmware to enable communication between the software and the stepper motors. This firmware serves as an interpreter for the G-code commands generated by the Universal G-code Sender, allowing the microcontroller to control and drive the stepper motors effectively.

With the combination of the Universal G-code Sender software and the GRBL firmware, precise movements of the stepper motors are achieved, facilitating the towing of the specimen at specific speeds within the tank. Table 1 provides a list of the files needed to create the open-source device.

**Table 1:** List of design files.

**Design file name**	**File type**	**Open source license**	**Location of the file**
SolidWorks folder	CAD	CC BY 4.0	https://doi.org/10.17632/k5y3ffzn3b.1
STL Folder	STL	CC BY 4.0	https://doi.org/10.17632/k5y3ffzn3b.1
DXF folder	DXF	CC BY 4.0	https://doi.org/10.17632/k5y3ffzn3b.1
GRBL firmware	Arduino script (.ino)	CC BY 4.0	https://doi.org/10.17632/k5y3ffzn3b.1
UGS software folder	Folder containing software for controlling system	CC BY 4.0	https://doi.org/10.17632/k5y3ffzn3b.1
Electronic schematic	KiCAD schematic and pdf	CC BY 4.0	https://doi.org/10.17632/k5y3ffzn3b.1
BOM	Spreadsheet (.xls)	CC BY 4.0	https://doi.org/10.17632/k5y3ffzn3b.1
Specimen samples	STL	CC BY 4.0	https://doi.org/10.17632/k5y3ffzn3b.1
Guide	pdf	CC BY 4.0	https://doi.org/10.17632/k5y3ffzn3b.1

### 3.5 SolidWorks folder

This folder contains 3D design files of the entire setup modelled in SolidWorks 2022. Within it are two sub folders –Control box and Gantry-Carriage.

### 3.6 STL folder

This folder contains the files that should be printed using a 3D printer. These parts must be printed individually to ensure finer prints.

### 3.7 DXF folder.

This folder contains files specifically designed for laser cutting using the laser cutter. Depending on the working area of the laser cutter available, it is possible to merge and cut all the files in the folder in a single run.

### 3.8 GRBL firmware

This zipped folder contains an Arduino file (grbl.ino) meant to be uploaded on the Arduino microcontroller to control the CNC machine.

### 3.9 UGS software folder

This folder contains files within which the main software for operating the CNC machine can be found. The main file is within the bin folder as ugsplatform for 32-bit machines and ugsplatform64 for 64-bit machines.

### 3.10 Electronic schematic

This folder contains the schematics for both circuit designs – the force measurement system and the control box.

### 3.11 BOM

This document includes a list of all the materials and components utilized in the system fabrication and the total cost involved.

### 3.12 Specimen samples

This folder contains the CAD and STL files of the test specimen samples that should be printed using a 3D printer.

### 3.13 Guide

This folder contains two portable document format (pdf) files – one instructional guide to wire the system and the other on how to use PIVLab software for flow visualization, as needed.

## 4 Bill of materials summary

A link to the BOM can be found in the design files table.

## 5 Build instructions

Given the relative complexity of this system, the build manual is organized into seven categories to provide detailed instructions for each component. These include gantry, carriage, gantry-carriage assembly, control box, tank, external wiring, and firmware configuration.

Prior to the commencement of the build, this quantity of parts should be readily available to make the process seamless.

Number of 3D printed parts: 37.

Number of Laser cut parts (5 mm plexiglass): 22.

Number of Laser cut parts (10 mm plexiglass): 4.

NB: If 10 mm plexiglass can’t be obtained, laser cut two pieces of 5 mm plexiglass and glue together for the respective part.

Table 2 shows the list of complete parts with quantities in bracket that ought to be 3D printed and laser cut before the build process commences. It is also helpful to group the parts to make the entire build process easier.

**Table 2:** Parts to be 3D printed and laser cut.

3D printed parts	Laser cut parts (5 mm plexiglass)	Laser cut parts (10 mm plexiglass)
**Gantry**: pulleyStand (2), motor bracket (1), XlimitSwitchHolder (1).	**Tank**: Tank-Length (2), Tank-base (2), Tank-width (2).	
**Carriage**: Slider (4), SupportForCameraPlatform (1), YLimitSwitchHolder (1), attachment_holder_camera (1), belt_Locker (2), BeltTopLock (2), CameraMount (1), CarriageEndMount (1), CarriageMotorMount (1), mount_box1 (1), mount_box2 (1), MountDown (1), MountTop (1), PlatformMerger (1).	**Carriage**: CameraVerticalPlatform (2), V-wheel platform (2), cameraCPlatform (1), CameraPlatform (1), dragChain mount (1), DragChainSupport2 (1), dragsupport 2.0 (1), ylimplatform (1).	**Carriage**: box_linker (2), Carriage_platform (2),
**Control box:** barrelJack holder (14).	**Control box:** back_face (1), Fan_face (1), Frame_face (1), Front_face (1), Port_face (1), Top_face (1).	

### 5.1 Gantry

**Fig. 2:**
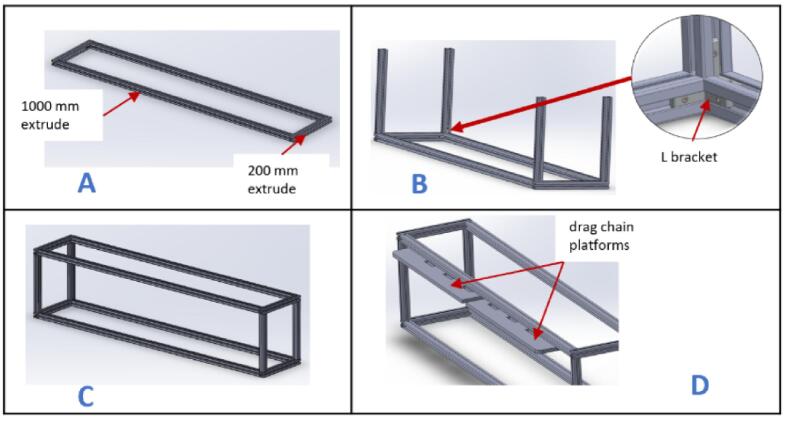
Key steps involved in building the gantry.
d. Screw each corner in place using an L bracket and a nut.
e. Place one 210 mm aluminum extrusion vertically on each corner and fix them in place using an L bracket (Fig. 2B).
f. Repeat steps a and b, and join it to the previous setup using L brackets (Fig. 2C).
g. Place the drag chain platforms beneath the gantry's top member and fasten them using 5 mm threaded screws and nuts (Fig. 2D).
h. Place a stepper motor into the motor bracket (motor bracket), screw it in place using four M3 x 10 screws, and attach a 5 mm bore pulley to the motor's shaft (Fig. 3A).

a. Have these laser cut parts: drag chain platforms (DragChainSupport2 and DragChainSupport2.0).
b. Have these 3D printed parts: (motor bracket), pulley stands (pulleyStand), and x-axis limit switch holder (XlimitSwitchHolder).
c. Cut two 1000 mm and two 200 mm 2020 aluminum extrusions and arrange them as shown in Fig. 2A.

**Fig. 3:**
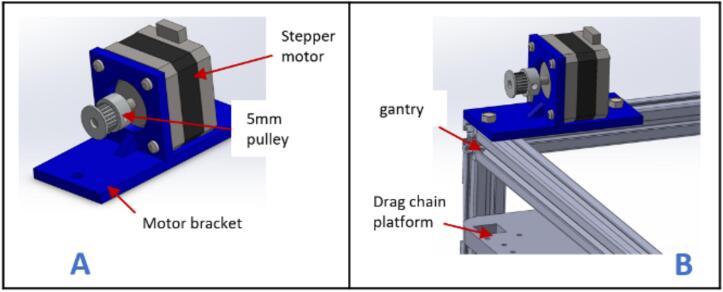
Assembling and fastening the X axis stepper motor on the gantry.
i. Position the stepper motor assembly at one corner of the gantry with the shaft facing outward (Fig. 3B) and secure it in place using two 5 mm threaded bolts and nuts.
j. Insert a 20 x 8 mm bearing into each pulley stand.
k. Place an 8 mm bore 20 T pulley on a 200 x 8 mm shaft. Do not tighten it yet.
l. Insert the shaft through the bearings and secure it in place using an Allen key (Fig. 4A).

**Fig. 4:**
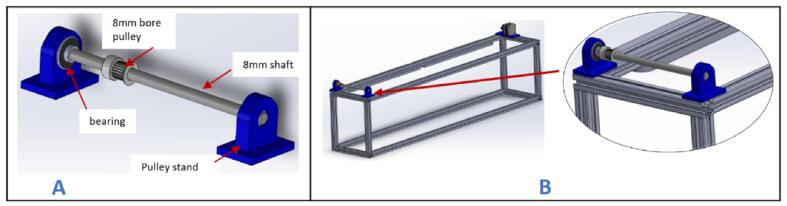
Mounting the pulley stands at the end of the gantry.
m. Slide the 8 mm bore 20 T pulley close to the left pulley stand and tighten it using an Allen key.
n. Position this new assembly on the opposite side of the gantry and fasten it in place using 5 mm threaded bolts and nuts (Fig. 4B).
o. Insert a wired limit switch into the X-axis limit switch holder.

**Fig. 5:**
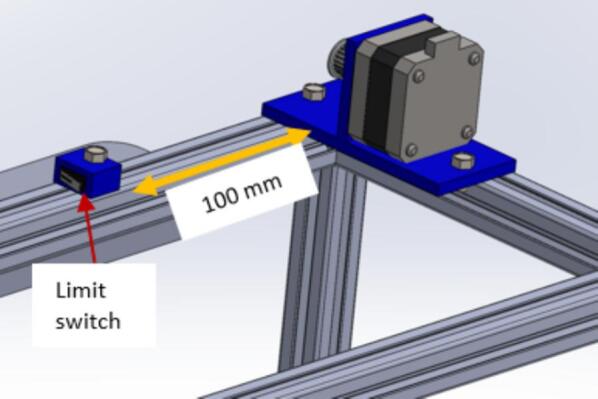
Mounting the X axis limit switch about 100 mm away from the X-axis stepper motor.

NB: Instructions for wiring the limit switches can be found in section 5.6.

p. Position the limit switch assembly on the gantry, 100 mm from the X-axis stepper motor. Secure it in place using a 5 mm threaded bolt and nut (Fig. 5).

### 5.2 Carriage

This section is sub-divided into five parts – Carriage holder 1.0, Carriage holder 2.0, Flow-visualization mount, V wheels assembly, and carriage assembly.

#### 5.2.1 Carriage holder 1.0

**Fig. 6:**
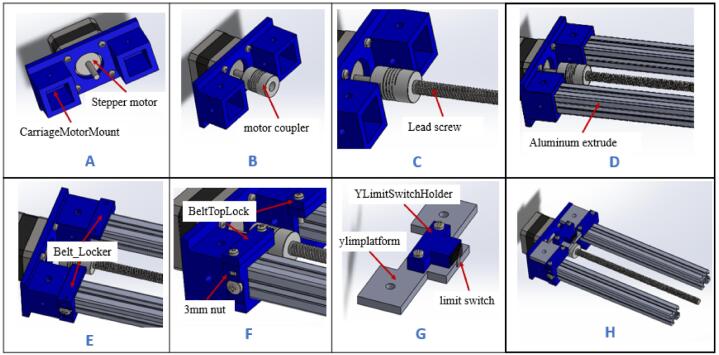
Assembling the carriage (part 1).
d. Attach a motor coupler to the shaft of the stepper motor and tighten using an Allen key (Fig. 6B).
e. Insert the 190 mm lead screw into motor coupler and secure it in place using Allen keys (Fig. 6C). The lead screw will not come at this given length, so you have to size it with a hacksaw.
f. Insert the two 200 mm 20x20 aluminum extrusions into (CarriageMotorMount) as shown in Fig. 6D.
g. Fix (belt_Locker) onto the extrusions but close to (CarriageMotorMount) and hold in place using M5 x 10 mm screws (Fig. 6E).
h. Insert a 3 mm nut in each slot of (belt_Locker) as shown in Fig. 6E, place (BeltTopLock) on top, and screw in place using M3 x 16 mm screw (Fig. 6F).
i. Insert a limit switch into (YLimitSwitchHolder). Make sure it is wired before inserting into the holder. Refer to guide document on how to wire the limit switch.
j. Place the limit switch assembly on (ylimplatform) and screw in placing using two M3 x 16 screws and nuts (Fig. 6G).
k. Place the assembly made on (CarriageMotorMount) as shown in Fig. 6H. Do not screw it in place for now as it should be a tight fit.

a. Have these 3D printed parts: (YLimitSwitchHolder), (CarriageMotorMount), (belt_Locker), and (BeltTopLock).
b. Have this laser cut part: (ylimplatform).
c. Attach the stepper motor to the (CarriageMotorMount) and hold it in place using four M3 x 16 mm screws (Fig. 6A).

#### 5.2.2 Carriage holder 2.0

**Fig. 7:**
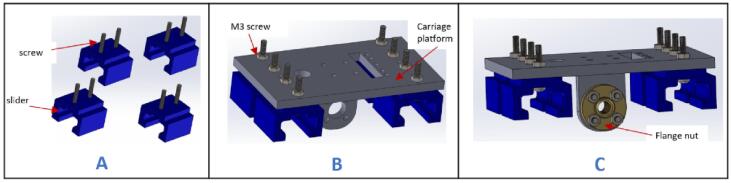
Assembly process for the carriage platform.
d. Place the carriage platform on the sliders and ensure that the screws pass through it. Fasten it in place using M3 nuts (Fig. 7B).
e. Insert a flange nut into the carriage platform and secure it using four M3 x 16 mm screws and nuts (Fig. 7C).
f. Pass the sliders through the aluminum extrudes (Fig. 8).

a. Have these 3D printed parts: (CarriageEndMount), sliders (slider), carriage platform (CarriagePlatform).
b. Have this laser cut part: drag chain mount (dragChain mount).
c. Insert two M3 x 20 mm screws in each of the four sliders (Fig. 7A).

**Fig. 8:**
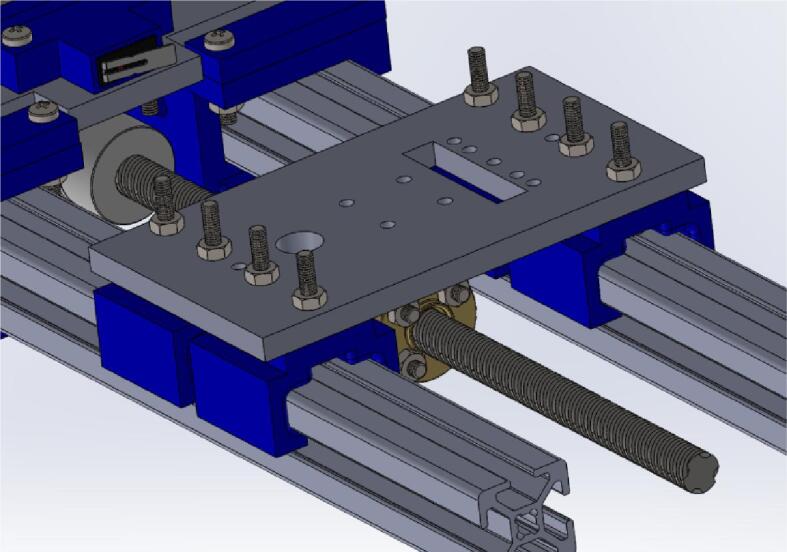
Mounting the carriage platform on the carriage.
g. Insert a 20 x 8 mm bearing into the 3D printed part (CarriageEndMount) (Fig. 9A).

**Fig. 9:**
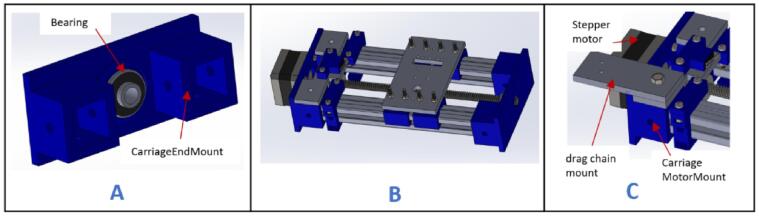
Assembling the carriage (part 2).
h. Insert the assembled part at the open end of the completed part from Carriage holder 1.0 (Fig. 9B).
i. Place the drag chain mount on the (CarriageMotorMount) and secure it in place using M5 x 10 mm bolt and nut (Fig. 9C).

#### 5.2.3 Flow-visualization mount

**Fig. 10:**
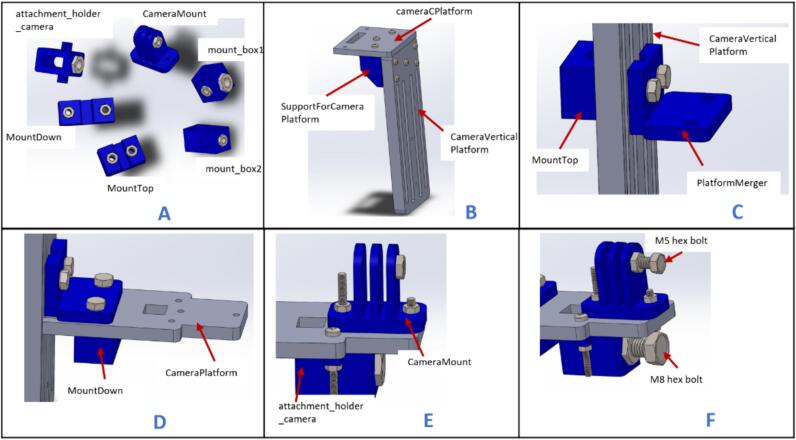
Assembly process for the flow-visualization mount.
d. Join (SupportForCameraPlatform), (CameraCPlatform), and (CameraVerticalPlatform) together using M3 x 16 mm screws and nuts (Fig. 10B).
e. Connect the (PlatformMerger) to (CameraVerticalPlatform) and secure it in place by screwing it onto (MountTop) using 5 mm bolts (Fig. 10C).
f. Align (CameraPlatform) directly beneath (PlatformMerger) and secure it in place by screwing it onto (MountDown) using 5 mm bolts (Fig. 10D).
g. Place (CameraMount) and (attachment_holder_camera) on top and beneath (CameraPlatform) and screw them in place using M3 x 16 screws (Fig. 10E).
h. Insert an M8 x 16 hex bolt and M5 x 16 mm hex bolts into the nuts of the (attachment_holder_camera) and (CameraMount) respectively (Fig. 10F).

a. Have these 3D printed parts: (CameraMount), (attachment_holder_camera), (PlatformMerger), (SupportForCameraPlatform), (MountTop), (MountDown), (Mount_box1), and (Mount_box2).
b. Have these laser cut pieces: (cameraCPlatform),(CameraPlatform), (CameraVerticaPlatform), and (box_linker).
c. Place 5 mm and 8 mm hexagon nuts in the above 3D printed parts as indicated in Fig. 10A.

**Fig. 11:**
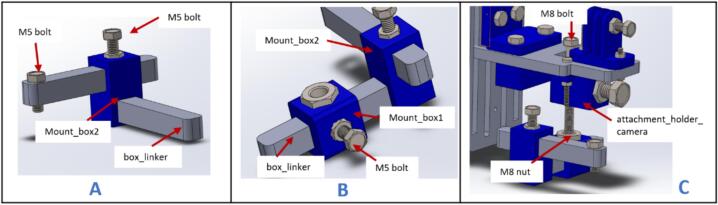
Assembling and inserting the specimen holder.
j. Slide (Mount_box1) onto the lower (box_linker) and fasten it in place using an M5 bolt (Fig. 11B).
k. Pass an M8 x 60 mm bolt through (attachment_holder_camera) and screw it onto the M8 nut on (Mount_box1) as shown in Fig. 11C.

NB: Do not fully tighten the bolts into the nuts.

i. Insert two (box_linker) parts into (Mount_box2). NB: The top part of (Mount_box2) is a through-hole, and the lower part isn't. Also, insert an M5 x 10 mm bolt in the upper (box_linker) as indicated in Fig. 11A. This bolt screws into the specimen.

##### 5.2.3.1 V wheels assembly

**Fig. 12:**
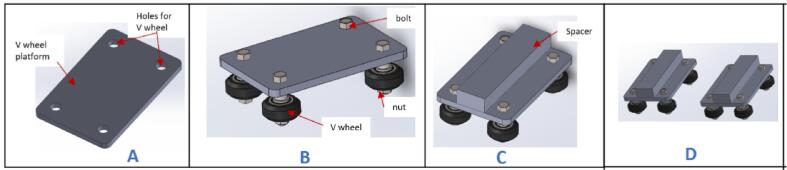
Assembling the V wheels.
c. Apply a weak-based glue to the surface of the spacer.
d. Stick the spacer to the V wheel platform using glue and ensure that the part is fixed at the central part of the platform (Fig. 12C).
e. Create another replica of this unit to have a total of two parts (Fig. 12D).

a. Have these laser cut pieces: V wheel platform (V-wheel platform) and spacers (Carriage_platform).
b. Take one part of the V wheel platform and insert four V wheels (Fig. 12B) into the holes. Fasten them in place using four M5 x 30 mm hex bolts and nuts.

##### 5.2.3.2 Carriage assembly

**Fig. 13:**
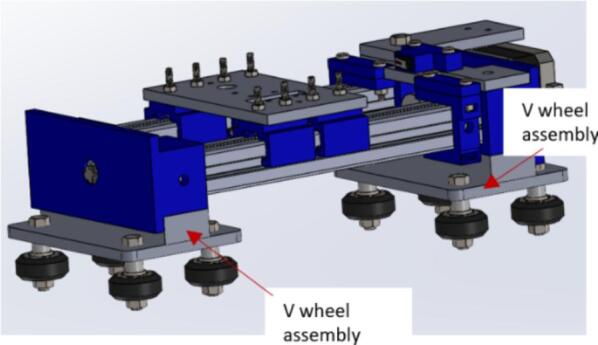
Final carriage assembly.

a. Apply glue on the surface of both V wheel assemblies and attach them beneath the carriage holders (CarriageEndMount and CarriageMotorMount), following the indications shown in Fig. 13.

### 5.3 Gantry-carriage assembly

**Fig. 14:**
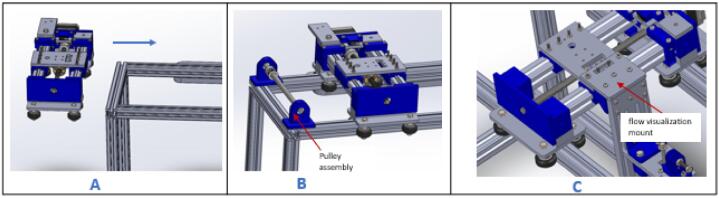
Mounting the carriage on the gantry.
c. Reattach the pulley assembly to the gantry and secure it using bolts (Fig. 14B).
d. Remove the two extreme nuts on the carriage platform facing the X-axis stepper motor. Position the flow visualization mount on it as indicated in Fig. 14C. Reinstall the nuts and tighten them securely.
e. Connect a 1-meter drag chain to the point indicated on the drag chain mount and secure it in place using M3 x 10 mm screws and nuts (Fig. 15).

a. Remove the pulley assembly from the gantry by unscrewing the bolts.
b. Slide the carriage through the top side of the gantry (Fig. 14A).

**Fig. 15:**
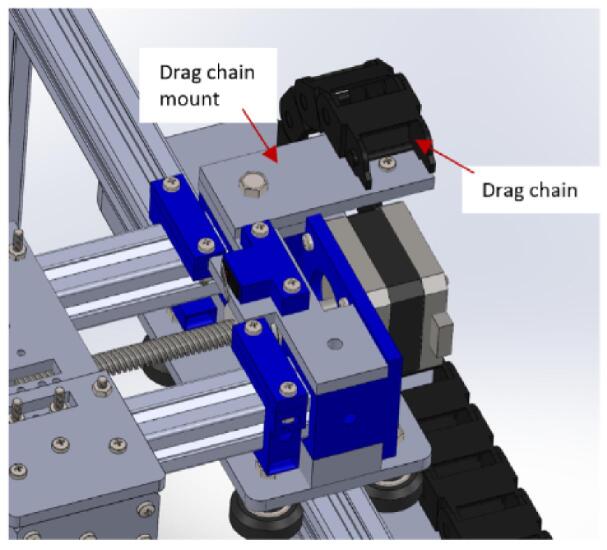
Mounting the drag chain on the carriage.
f. Cut approximately 1800 mm length of the timing belt.
g. Unscrew (BeltTopLock) from (belt_Locker) (Fig. 16A).

**Fig. 16:**
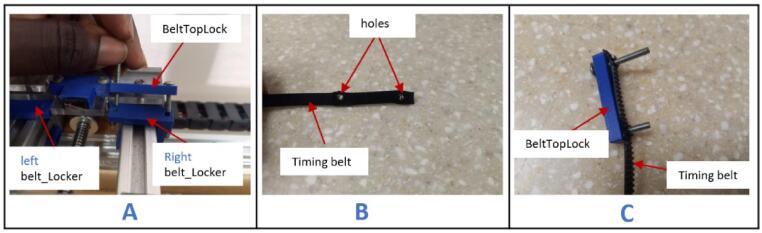
Mounting the timing belt (part 1).
h. Create two holes, 30 mm apart, at both ends of the timing belt (Fig. 16B).
i. Pass the screws from the (BeltTopLock) through the holes in the belt (Fig. 16C) and fasten it on the right side of the (belt_Locker).
j. Pass the timing belt over the pulley on the X-axis stepper motor (Fig. 17A), through the channels beneath the right (belt_Locker) (Fig. 17B) and left (belt_locker) (Fig. 17C).

**Fig. 17:**
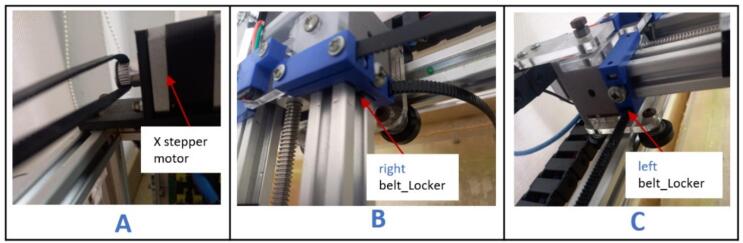
Mounting the timing belt (part 2).
k. Continue to pass the timing belt around the pulley on the pulley assembly (Fig. 18A) and screw the end of it on the left (belt_locker) (Fig. 18B).

**Fig. 18:**
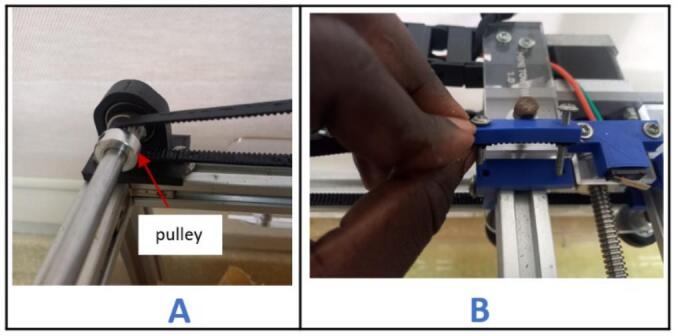
Mounting the timing belt (part 3).

### 5.4 Control box

This section is sub-divided into three parts – Frame fabrication, Component arrangement, and Control box assembly.

#### 5.4.1 Frame fabrication

**Fig. 19:**
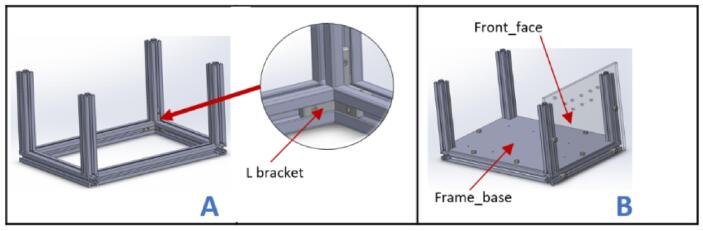
Control box assembly (part 1).
c. Place Frame_base and Front_face at the bottom on the face of the frame built as shown in Fig. 19B and fasten them with 5 mm bolts and nuts.

a. Have these laser cut pieces: back_face, fan_face, Frame_base, Front_face, Port_face, and Top_face.
b. Take two 130 mm, 230 mm, and 190 mm each of 2020 aluminum extrusions and arrange them as shown in Fig. 19A. Secure each joint with an L bracket.

#### 5.4.2 Component arrangement

**Fig. 20:**
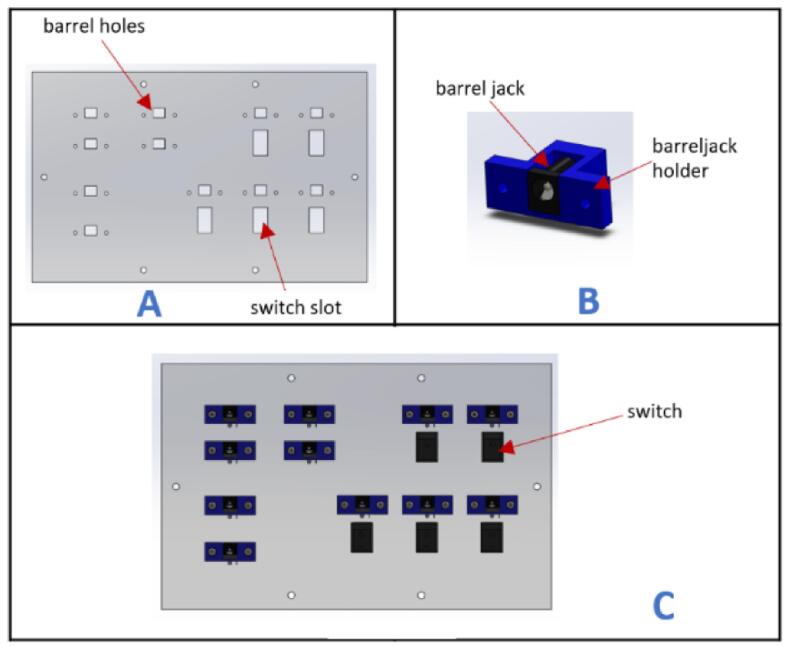
Port face of control box assembly.
c. Insert one barrel jack into the (barreljack holder) (Fig. 20B). Create fourteen replicas of this barrel jack and (barrelJack holder) assembly. Only eleven pieces of this assembly will be used on this face. Refer to the Wiring instructional guide for procedure on how to wire the barrel jack. Also make sure to wire the barrel jack before inserting it into its holder.
a. Fig. 21 illustrates the designations for the ports on the port face. Please note that the unlabeled ports are optional and can be utilized to accommodate other functionalities based on the user's preferences.

a. Have this 3D printed part: (barrelJack holder).
b. Take (Port_face) and insert switches into each switch slot. Five switches should be inserted for this face (Fig. 20A).

**Fig. 21:**
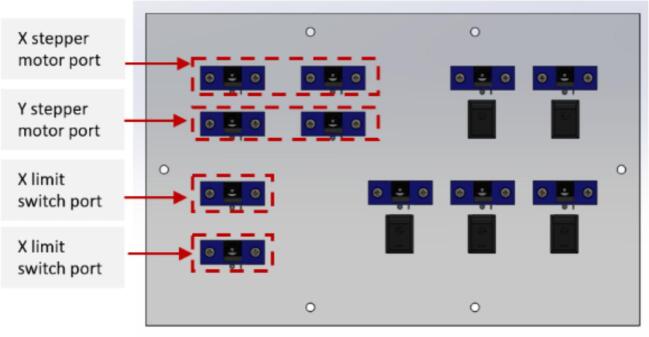
Port face on the control box.
d. Take the (fan_face) and attach the Small fan, Big fan, two switches, and one barrel jack assembly to it using M3x16 mm screws (Fig. 22A).

**Fig. 22:**
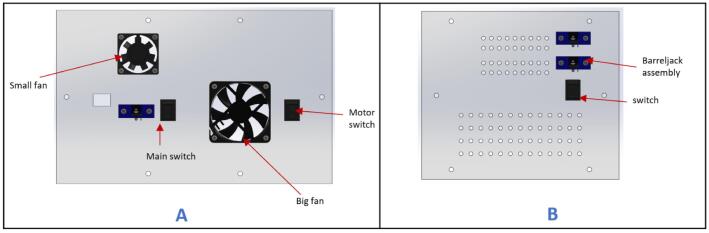
Fan face of the control box assembly.
e. Take (Top_face) and insert the Emergency Stop button.
f. Take (back_face) and insert two-barrel jack assemblies with M3x16 mm screws and one switch (Fig. 22B). Please note that the components attached on this face are optional.
g. Place two stepper motor drivers (Lead shine DM556), two buck converters (DC-DC Buck converter), and four spacers at the specified locations on the (Frame_base) part (Fig. 23).

**Fig. 23:**
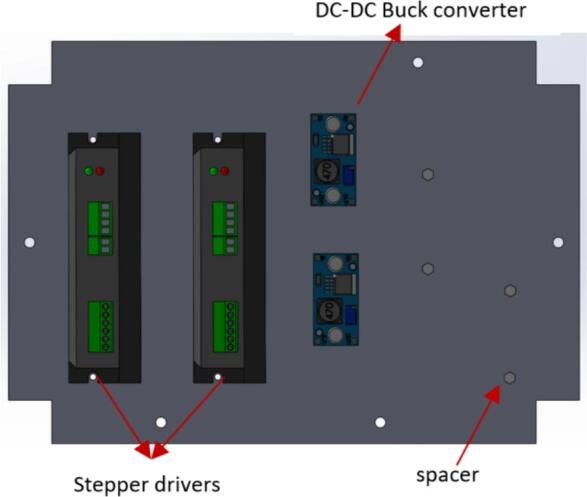
Placement of motor drivers and buck converters on the frame base.
h. Arrange the Arduino uno (ARDUINO UNO R3), two [LM7812-Transistor], two [L7805-transistor], a 5 mm LED, and two optocouplers on a perf board (Perforated_board) (Fig. 24A).

**Fig. 24:**
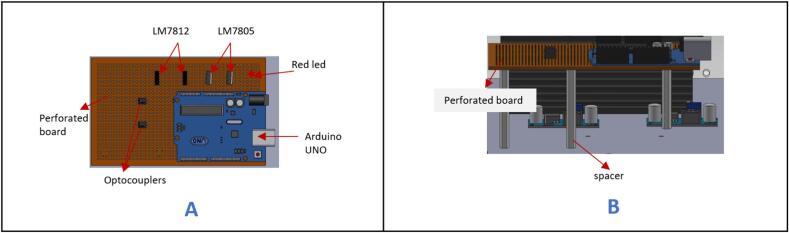
Placement of electronics on the perforated board.
i. Follow the wiring instructional guide provided in the Electronic Schematic folder to connect the various electronic components together using wires and solder. Kindly note that some connections are optional and are indicated in the guide.
j. Use spacers to elevate the perforated board and secure it on the specified holes indicated in Fig. 24B.

#### 5.4.3 Control box assembly

**Fig. 25:**
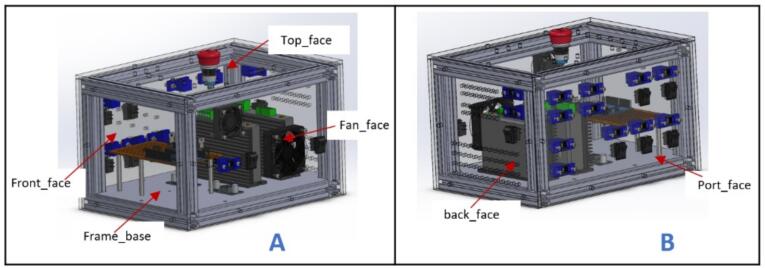
Control box final assembly.

a. Position the remaining faces on their respective sides of the control box frame (Fig. 25) and secure them in place using M5 x 10 mm bolts and nuts.

### 5.5 Tank

a. Have these laser cut parts: Tank-base, Tank-Length, and Tank-width.

**Fig. 26:**
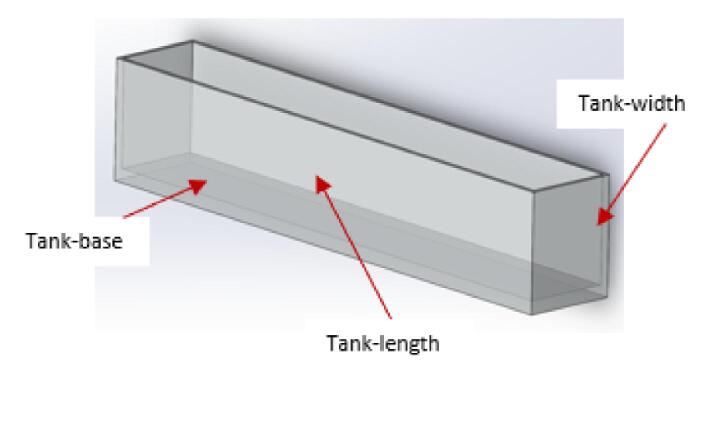
Parts of the assembled tank.
c. Seal the inner joints of the tank using silicone glue and leave it to dry for 48 h.

NB: Depending on the source of your plexiglass, it may come with a paper or rubber liner. This liner can be removed after laser cutting or left in place. Its main purpose is to prevent scratches on the plexiglass during handling and keeps it in good condition until you use it.

b. Arrange the parts as shown in Fig. 26. Apply some superglue at the corners to temporarily hold the parts in position.

### 5.6 External wiring

#### 5.6.1 X-axis limit switch

**Fig. 27:**
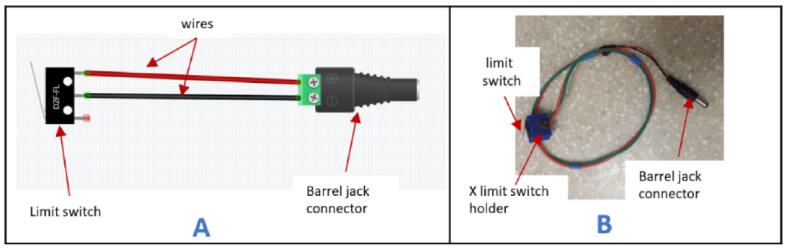
Wiring of the X-axis limit switch.
e. Connect the barrel jack connector to the control box on the port face designated for the X-axis limit switch.

a. Take the X-axis limit switch holder (XLimitSwitchHolder).
b. Solder a wire, approximately 300 mm in length, to the Common (C) and Normally Open (NO) terminals on the limit switch, respectively.
c. Insert the limit switch into the X-axis limit switch holder.
d. Connect the other ends of the wire to a barrel jack connector (Fig. 27).

#### 5.6.2 Y-axis limit switch

a. Take the Y-axis limit switch holder (YLimitSwitchHolder).
b. Solder a wire, approximately 1300 mm in length, to the Common (C) and Normally Open (NO) terminals on the limit switch, respectively.
c. Insert the limit switch into the Y-axis limit switch holder.
d. Connect the other ends of the wires to a barrel jack connector, as shown in Fig. 27B.
e. Pass the cables through the drag chain.
f. Connect the barrel jack connector to the control box on the port face designated for the Y-axis limit switch.

#### 5.6.3 X-axis stepper motor wiring

**Fig. 28:**
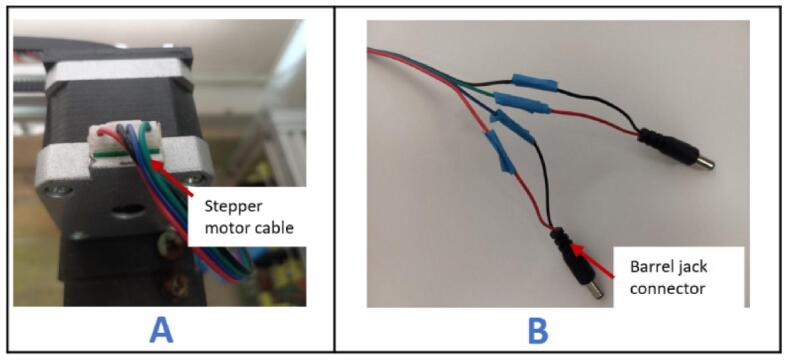
Wiring of the X-axis stepper motor. A- stepper cable inserted in stepper motor, B – ends of stepper cable connected to barrel jack connectors.
b. Strip the cables and solder the A+1 (red wire) and A-1 (blue wire) to one barrel jack connector, and the B+1 (green wire) and B-1 (black wire) to a separate barrel jack connector (Fig. 28B).
c. Connect both barrel jack connectors to the control box on the port face designated for the X-axis stepper motor.

a. Insert a 300 mm stepper motor cable into the X-axis stepper motor (Fig. 28A).

#### 5.6.4 Y stepper motor wiring

a. Insert a 1200 mm stepper motor cable into the Y-axis stepper motor.
b. Strip the cables and solder the A+2 (red wire) and A-2 (blue wire) to a barrel jack connector, and the B+2 (green wire) and B-2 (black wire) to a separate barrel jack connector.
c. Pass the cables through the drag chain.
d. Connect both barrel jack connectors to the control box on the port face designated for the Y-axis stepper motor.

### 5.7 Uploading firmware

**Fig. 29:**
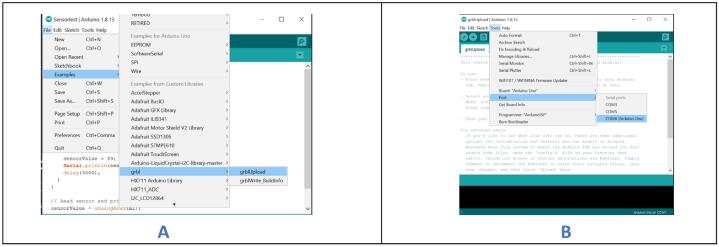
Uploading GRBL onto the Arduino board.
h. Connect the Arduino to your PC using a USB B cable.
i. In the Arduino IDE, navigate to “Tools,” then “Port,” and select the appropriate port number from the list (Fig. 29B).
j. Click the upload button in the Arduino IDE to send the code into the Arduino. This will install the GRBL firmware on Arduino.

a. Download Arduino IDE from https://www.arduino.cc/en/software.
b. Download GRBL from the design file folder and unzip it.
c. Locate GRBL folder and copy it.
d. Go to the section on your PC where Arduino was installed.
e. Open the “Libraries” folder within the Arduino folder.
f. Paste the GRBL folder into the “Libraries” folder and close the window.
g. Launch the Arduino IDE and navigate to “File,” then “Examples,” and select “grblUpload” from the list (Fig. 29A). Ensure that you do not edit the file.

### 5.8 UGS configuration

**Fig. 30:**
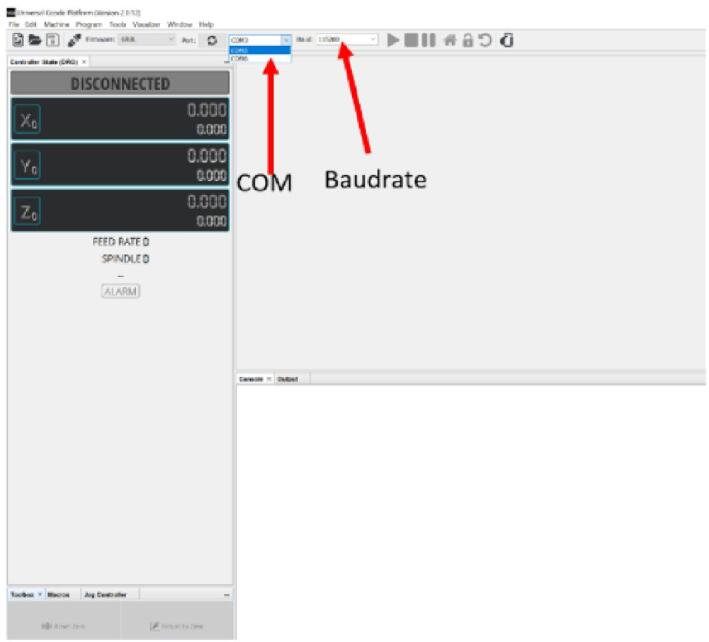
Configuration of Universal Gcode Sender.

a. Download Universal G-code Sender from the design file folder.
b. After downloading, unzip the file and navigate to the bin folder.
c. In the bin folder, open either “ugsplatform” for 32-bit machines or “ugsplatform64″ for 64-bit machines.
d. Inside Universal G-code Sender, select the appropriate COM port number and set your baud rate to 115,200 (Fig. 30). This configuration ensures the right communication with the Arduino with GRBL firmware installed.

## 6 Operation instructions

### 6.1 Powering system

**Fig. 31:**
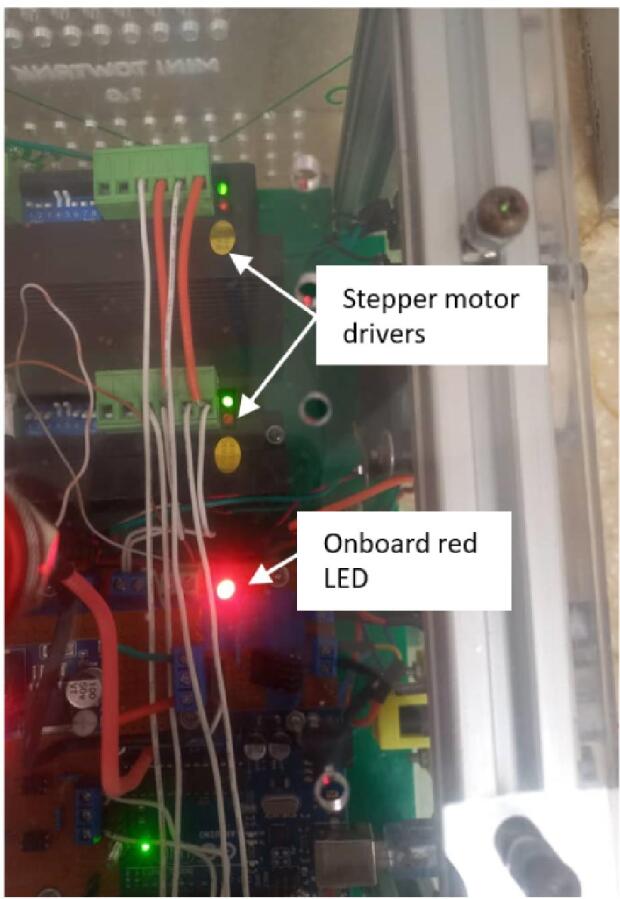
Onboard red LED indicator in the control box. (For interpretation of the references to color in this figure legend, the reader is referred to the web version of this article.)
c. Turn on the stepper drivers by pressing the “Motor” switch. This should turn on the big fan and power the stepper motor drivers.

a. Connect a 24 V 3A power source to the Power inlet port labeled “Supply port” on the Control Box.
b. Turn on the unit by pressing the “Main switch” This action should turn on the small fan and light up the onboard red LED (Fig. 31).

**Fig. 32:**
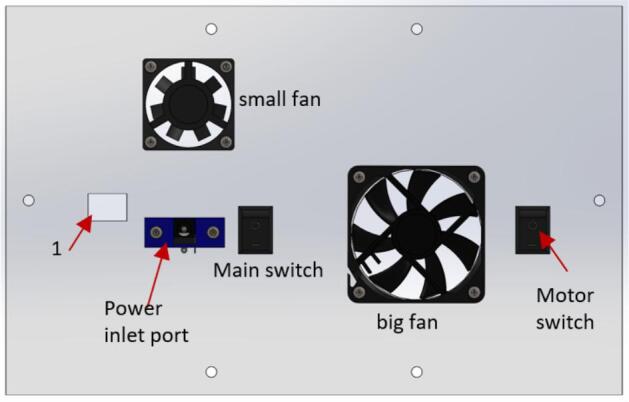
Parts of the fan face on the control box.

The hole marked as “1″ in Fig. 32 is the entry port for the USB cable that connects to the Arduino microcontroller.

### 6.2 Preparation of tank

**Fig. 33:**
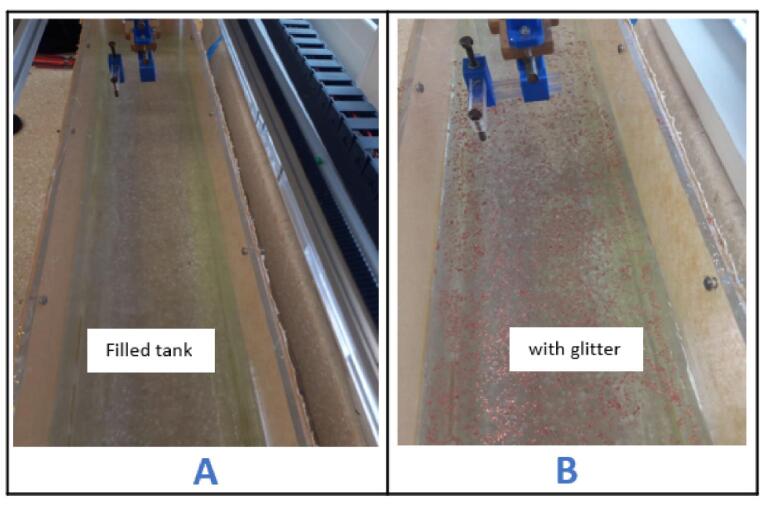
Filling and seeding the tank with water and glitter.
b. Sprinkle nail glitter over the water's surface to serve as the tracer particles which will enable the visualization of flow patterns (Fig. 33B).

a. Fill the tank with water until it reaches about 4/5 of its total volume (Fig. 33A).

### 6.3 Setting up and calibrating the system

**Fig. 34:**
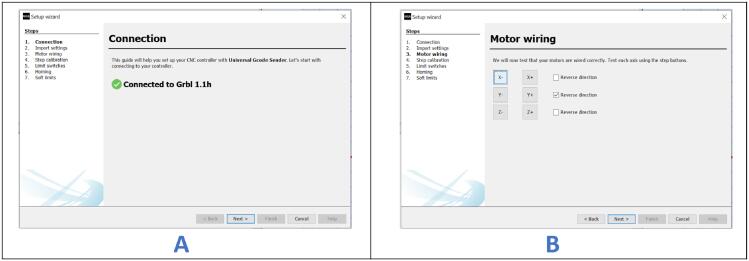
Calibrating the setup (part 1).
d. Click “Next” until the “Motor wiring” screen appears (Fig. 34B).
e. On the “Motor wiring” screen, click on “X-”, “X+”, “Y-”, or “Y+” to test if the motors move in the correct direction. If any motor moves in the wrong direction, check the box next to the respective button to change the direction. Click “Next” to proceed to the “Step calibration” section.
f. Mark an origin point on the carriage using a pencil on your CNC setup.
g. On the “Step Calibration” window (Fig. 35A), click on “X+” to increase the value highlighted as “1″ and move the carriage from 0 mm to 10 mm. This should jog the carriage over a specified distance. Mark the new position and record the distance covered.

This process is performed during the initial setup of the system.

a. Turn on the system.
b. Open Universal G-code Sender (UGS) and select the appropriate COM port number and baud rate.
c. On the toolbar, click “Machine,” then select “Setup wizard.” A small dialogue box (Fig. 34A) will appear.

**Fig. 35:**
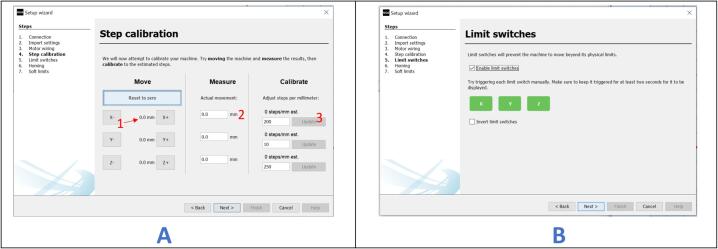
Calibrating the setup (part 2).
h. Record this value in the edit field marked as “2″.
i. Click on the “Update” button (highlighted as “3″) to store the new value into the firmware.
j. Repeat the process for the Y-axis. Click “Next” to proceed to the “Limit switches” window (Fig. 35B).
k. Check the “Enable limit switches” box to activate the limit switches.
l. Press the X-axis limit switch for at least two seconds. This should change the appearance of the “X” icon from green to red.
m. Repeat the process for the Y-axis limit switch. Click “Next” to proceed to the “Homing” window (Fig. 36). NB: If the icon doesn't change appearance after triggering the limit switch, check the wiring of the limit switch. Only proceed if both limit switches work properly.

**Fig. 36:**
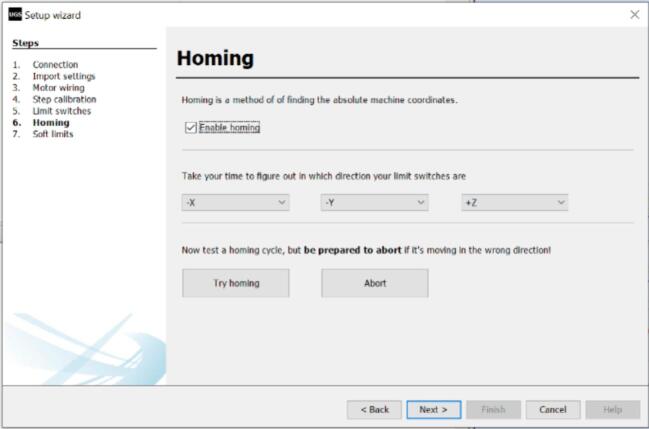
Calibrating the setup (Part 3).
n. Click on “Try Homing” to begin the Homing cycle. This should move both axes of the CNC setup until they reach their origin position close to the limit switches. Click on “Abort” if there isn't a successful homing operation.
o. Click on “Next” to proceed to the “Soft limits” window (Fig. 37).

**Fig. 37:**
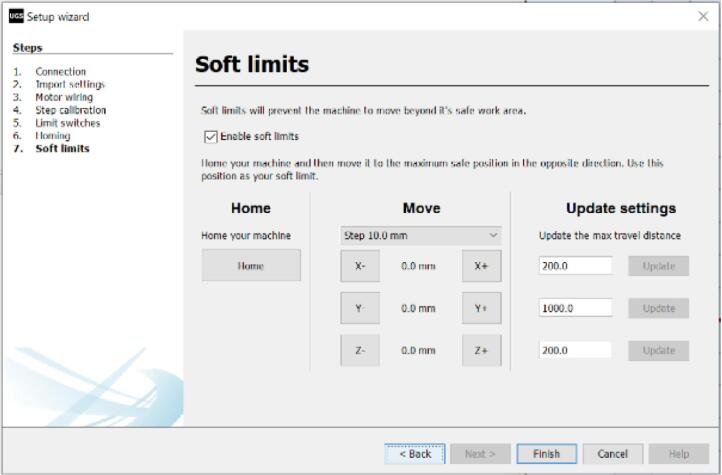
Calibrating the setup (Part 4).
p. Check the “Enable soft limit” box. Change the parameters under “Update settings” to set your maximum travel distance for the X and Y axes. This will prevent the CNC setup from moving beyond the specified limits during operation.
q. Click on “Finish”.

### 6.4 Mounting specimen

**Fig. 38:**
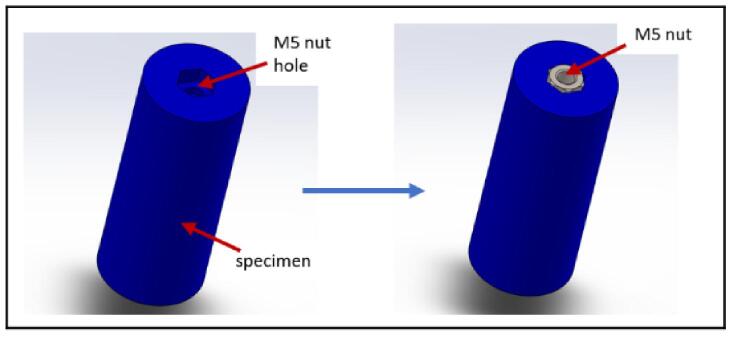
Cylindrical test specimen.
c. Attach the specimen to the CNC setup by screwing the M5 x 10 mm bolt on the upper (box_linker) into the M5 nut on the specimen (Fig. 39). This will securely hold the sample for testing or observation during towing tank experiments.

a. 3D print any of the test specimens from the Specimen samples folder. For example, “Cylinder”.
b. Place a 5 mm nut in the hole marked in Fig. 38 and secure it in place with glue.

**Fig. 39:**
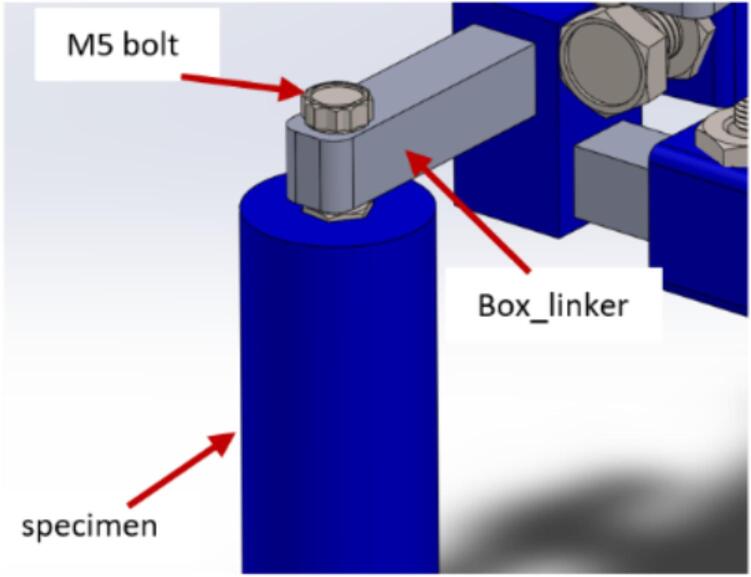
Mounting test specimen on mount.

### 6.5 Mounting camera

**Fig. 40:**
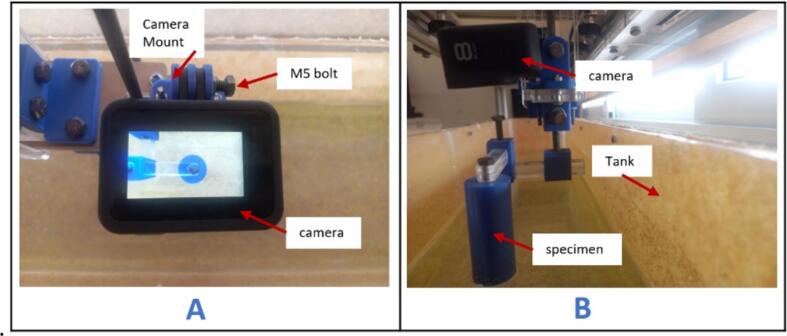
Mounting the camera on the mount.
b. Tilt the camera until the lens is facing downwards.
c. Lock the camera in place using the M5 x 10 mm bolt.

a. Slide the camera (GoPro Hero 8) through the (CameraMount) as indicated in Fig. 40A.

Fig. 40B provides a side view of the setup, showing the securely mounted camera and specimen.

### 6.6 Jog/Move system

The System can be jogged using either of two methods:

#### 6.6.1 Jog Controller Method

**Fig. 41:**
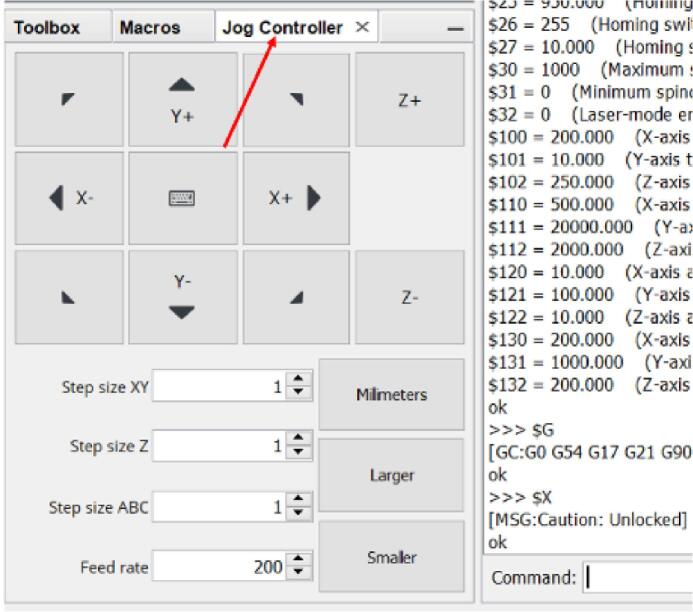
Jogging interface on Universal Gcode Sender.
b. Edit the “Step size XY” and/or “Feed rate” parameters to suit your specifications.
c. Click “X-”, “X+”, “Y-”, or “Y+” to move axis with specified parameters.

a. On the main screen of UGS, navigate to the “Jog Controller” tab (Fig. 41).

#### 6.6.2 Command line Method

**Fig. 42:**
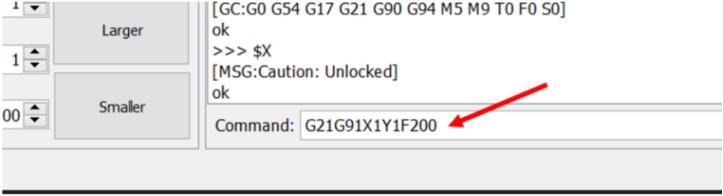
Jogging command to move X axis by 20 mm at a speed of 200 mm/s.

a. Using the syntax highlighted before, jog the carriage at the specified speed and distance by entering the command in the command line (Fig. 42).

G21G91X?F? – Jogging only X axis. Replace “?” with set distance and speed respectively.

G21G91Y?F? − Jogging only X axis. Replace first and second “?” with set distance and speed respectively.

G21G91X?Y?F? − Jogging both axis at the same time. Replace first, second, and third “?” with set end distances for X axis, Y axis, and speed.

For example:

G21G91X20F200

This will jog the X axis by 20 mm at a speed of 200 mm/s.

### 6.7 Emergency

**Fig. 43:**
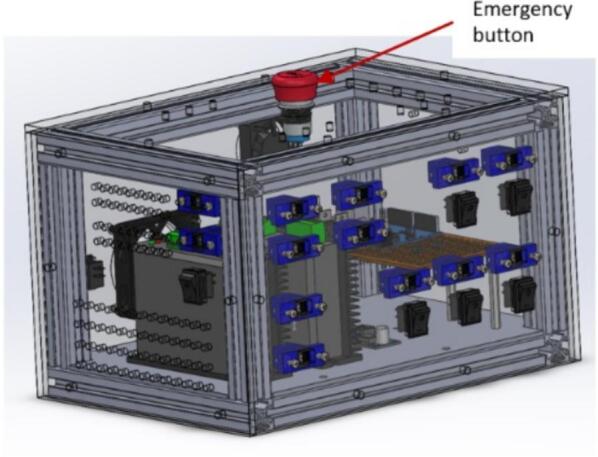
Emergency button on the control box.
b. If necessary, remove the USB cable from the PC or microcontroller. This disconnects communication between the control software and the CNC setup, preventing further commands or movements.
c. Power off the motors by switching off either the main switch or the motors switch on the control box. This will deactivate the stepper motors and stop any movement on the CNC setup.

If the system moves beyond its specified boundaries or fails to accept a jog command, you can use any of these outlined points to halt the process:

a. In case of an emergency, press the emergency button located on top of the control box (Fig. 43). Remember to release the switch before conducting another jogging or homing operation.

### 6.8 Running Experiment

a. Mount both camera and specimen of choice.
b. Set the camera to video mode and start recording.
c. Jog the system at a preferred speed in the x-axis.
d. Stop the video after the jog is complete.
e. Post process video in PIVLAB, MATLAB or any fluids processing software.

### 6.9 Turning off the system

a. Turn off the “Motor switch”.
b. Turn off the “Main switch”.
c. Turn off the power supply unit.

## 7 Validation and characterization

### 7.1 Axes’ motion accuracy test

To validate that both axes were properly calibrated, the system was jogged by 100 mm from its origin in both axes and the percent difference from the actual was found.

#### 7.1.1 X axis position accuracy test

**Fig. 44:**
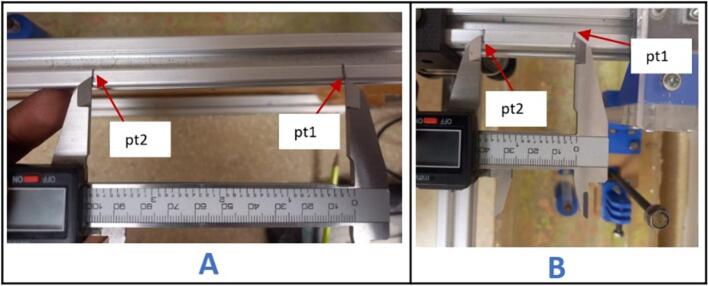
X axis position accuracy test. A- marking positions on the gantry, B- marking positions on the carriage.
b. The X axis was jogged by 100 mm in the Universal G-code Sender (UGS) software.
c. The final position was marked (pt2) after the jog was completed.

a. A point on the gantry close to the carriage was marked – pt1 (Fig. 44A).

Here are the results:

Actual distance: 100 mm.

Measured distance: 101 mm.

Percentage error:.$\frac{\left|Actualdistance-measureddistance\right|}{\mathrm{Actualdistance}}x100\%$

=.$\frac{\left|100mm-101mm\right|}{100mm}x100\%$

= 1 %.

#### 7.1.2 Y axis position accuracy test

An accuracy test similar to that in 7.1.1 was conducted on this axis but with a set distance of 40 mm, since the available span of motion is smaller in this direction. An initial point pt1, was marked, carriage was jogged by 40 mm, and the final point marked as pt2 as shown in Fig. 44B. Here are the results:

Actual distance: 40 mm.

Measured distance:40 mm.

Percentage error:.$\frac{\mathrm{Actualdistance}-measureddistance}{\mathrm{Actualdistance}}x100\%$

=.$\frac{\left|40mm-40mm\right|}{40mm}x100\%$

= 0 %.

With this level of accuracy, this CNC system can effectively execute motion profiles that it is programmed to perform.

### 7.2 Axes’ velocity test

To validate the velocity of both axes, a simple experiment was conducted.

#### 7.2.1 X axis velocity test

Three pointers were marked on the X axis along the gantry. The first pointer was 100 mm from the carriage when homed, the second was 200 mm from the first pointer, and the third was also 200 mm from the second pointer. For the first run, the time taken for the carriage to move from the first pointer to the second was recorded. For the second run, the time taken for the carriage to move from the first pointer to the third pointer was recorded. Each run was performed twice and averaged. The results are shown in Table 3.

**Table 3:** X axis velocity results.

Distance/mm	$t_{1}/s$	$t_{2}/s$	$t_{\mathrm{avg}}/s$	Velocity/mm/s	Percent error/%
200	25.18	24.90	25.04	7.99	0.13
400	49.90	50.03	49.97	8.01	0.13

Test speed = 480mm/min (.8mm/s)

From Table 3 above, the X-axis velocity recorded is precise with an accuracy of 99.87 %.

#### 7.2.2 Y axis velocity test

A similar procedure was conducted for the Y-axis along the carriage but using only two pointers due to its length. The first pointer was 20 mm from the carriage platform when homed. The second pointer was 50 mm from the first pointer. The time taken for the carriage platform to move from the first pointer to the second was recorded. This was performed twice and averaged. The results are shown in Table 4.

**Table 4:** Y-axis velocity test results.

Distance/mm	$t_{1}/s$	$t_{2}/s$	$t_{\mathrm{avg}}/s$	Velocity/mm/s	Percent error/%
50	10.10	10.30	10.20	4.90	2.00

Test speed = 300mm/min (.5mm/s)

From Table 4 above, the Y-axis velocity has an accuracy of 98 %.

### 7.3 Flow visualization tests

The next step was to test the system’s ability to visualize relevant flow features, such as wakes generated behind a body being towed at specific speeds.

Two test objects were selected for use in this testing. Both are bluff bodies – a circular cylinder and cuboid of dimensions, 25 mm in diameter and 25 mm x 10 mm respectively. Each was tested at three different Reynolds numbers, which was achieved by varying the towing speed. Images captured from the flow around the cylinder and cuboid were analyzed in MATLAB (PIVLab software) to generate the velocity fields. This is demonstrated in two sets of experiments, one set for each of the test objects. Key flow features were sought out in the results, such as identifying the stagnation point and velocity deficit in the wake.

*Experiment 1: Towing specimen 1 (circular cylinder) at Reynolds numbers of 319, 955, and 1,900.*

The following are a series of images showing first the raw image captured by the camera, in which one sees the glitter on the surface of the water and the top view of the test specimen and mount. Second is the processed image generated by the PIV software, in which one sees the velocity vectors overlaid on the image. Third is the final processed image in which one sees the velocity values overlaid in contour form on top of the vector field. In all cases, the flow direction is from right to left.

**Fig. 45:**
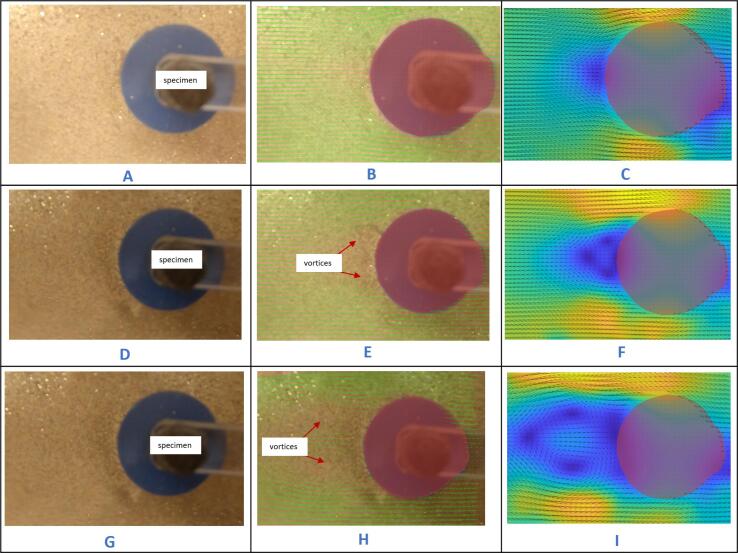
Raw and processed images of specimen towed at a Re of 319, 955, and 1,900. A- raw image of cylinder towed at Re of 319, B- mean velocity vector plot, C- color plot of specimen at Re of 319, D- raw image of cylinder towed at Re of 955, E- mean velocity vector plot, F- color plot of specimen at Re of 955, G- raw image of cylinder towed at Re of 1,900, H- mean velocity vector plot, I- color map of specimen at Re of 1,900.

Shown in Fig. 45B and C are processed images of a cylinder (Re = 319) towed within the tank. Fig. 45A is a raw image captured by the camera. Shown in Fig. 45E and F below are processed images of a cylinder (Re = 955) towed within the tank. Fig. 45D is a raw image captured by the camera. Shown in Fig. 45H and I below are processed images of a cylinder (Re = 1,900) towed within the tank. Fig. 45G is a raw image captured by the camera.

These results reveal that the system is able to capture expected features in the various flow fields. The stagnation point is visible in blue on the right side of the cylinder. The velocity deficit (in blue) in the wake grows as the Reynolds number increases. The flow around the cylinder remains relatively undisturbed at slow speeds, showing a remarkably smooth profile (Fig. 45C) with no noticeable vortices generated. As the Reynolds number increases slightly, a transition subtly begins taking place as small vortices emerge in the cylinder's wake (Fig. 45F). The flow continues to evolve as the Reynolds number increases further, with the generation of even more pronounced vortices in the wake generated (Fig. 45I). This progression agrees with the long-established characteristics of flow around a circular cylinder starting as a regime of unseparated flow at very low Reynolds numbers, then at a slightly higher Reynolds number transitioning to one that develops a pair of fixed vortices in the wake, before transitioning at a higher Reynolds number to one that generates a von Kármán vortex wake which is made of vortices being deposited in the wake in an alternating manner.

*Experiment 2: Towing specimen 2 (cuboid) at Reynolds numbers of 955 and 382.*

The second set of results were generated from towing the cuboid down the tank. The towing speed was held constant, but the orientation of the object was rotated by 90 degrees, in effect causing the same object to change from a bluff body to a streamlined one. The Reynolds number was calculated based on the cross-flow dimension of the object.

**Fig. 46:**
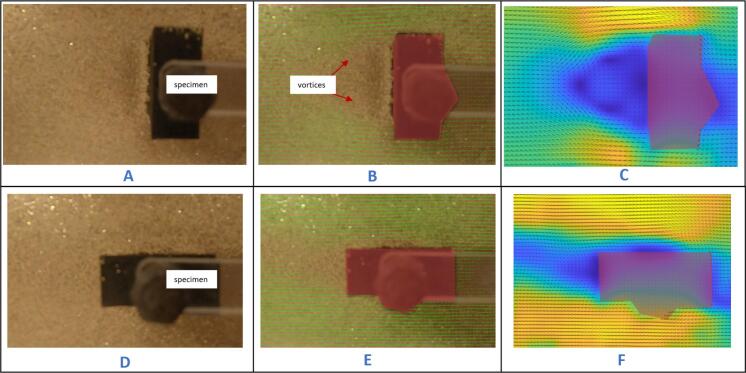
Raw and processed images of the specimen towed at a Re of 955 and 382. A- raw image of cuboid towed at Re of 955, B- mean velocity vector plot, C- color plot of specimen at Re of 955, D- raw image of cuboid towed at Re of 382, E- mean velocity vector plot, F- color plot of specimen at Re of 382.

Shown in Fig. 46B and Fig. 46C below are processed images of the specimen (Re = 955) towed within the tank. Fig. 46A is a raw image captured by the camera. Shown in Fig. 46E and F below are processed images of a cuboid (Re = 382) towed within the tank. Fig. 46A is a raw image captured by the camera.

The experimental results from the cuboid being towed at a constant speed demonstrate the significant influence of the area facing the flow on what is developed. When the specimen was oriented such that its longer side faced the direction of the moving stream (as shown in Fig. 46A), it generated a larger wake with noticeable vortices trailing behind it. However, when the orientation was changed, causing the shorter side to face the oncoming stream (Fig. 46D), it produced a smaller wake with no visible vortices. This illustrates that the size and features of the wakes generated by a bluff body in motion through a fluid can significantly change when the object's characteristic length and orientation are altered while the speed remains constant.

Understanding these concepts plays a significant role in fluid dynamics and has implications for various engineering applications. Researchers and students often study vortices and wakes to gain insights into flow behavior, lift and drag forces, and the interactions between bodies and the surrounding fluid. Also, analyzing these phenomena in towing tanks can aid in improving the designs of hydrodynamic vessels such as submarines.

### 7.4 System specifications

Highlighted below are the specifications of the unit:

- Power supply: 24 V 3A.
- Working length: less than 650 mm.
- Max allowable speed: 160 mm/s.
- Specimen loading time: less than 2 min.
- Jog accuracy for X and Y axes: 99 % and 100 %, respectively.
- Speed accuracy for X and Y axes: 99.87 % for X-axis for 98 % for Y axis.

*NB: The accuracy of the jog and speed can be improved by going through the calibration steps highlighted in* section 6.3 *.*

- Designed to hold only GoPro Hero cameras.
- Mount on specimen holder limits the variety of specimens that can be attached to the unit.

### 7.5 Future works

The system, as currently designed, focuses solely on observing surface phenomena. A laser module can be integrated with the unit to illuminate tracer particles dispersed into the tank to enhance its capabilities. By coupling the laser module and integrating neutrally buoyant tracer particles, a complete Particle Image Velocimetry (PIV) setup can be established. This addition will capture and analyze fluid flow dynamics in greater detail. The tracer particles will act as markers, allowing researchers to track the movement and behavior of the fluid throughout the towing tank. As a result, a more comprehensive understanding of surface and internal flow phenomena can be achieved, making the system a valuable tool for advanced fluid dynamics research.

### CRediT authorship contribution statement

**Jeremiah Takyi:** Writing – original draft, Visualization, Validation, Software, Methodology, Conceptualization. **Heather R. Beem:** Writing – review & editing, Visualization, Supervision, Resources, Funding acquisition, Conceptualization.

## Declaration of competing interest

The authors declare that they have no known competing financial interests or personal relationships that could have appeared to influence the work reported in this paper.

## References

[1] B. Carey, A one-of-a-kind wind tunnel for birds paves the way for better drones | Stanford University School of Engineering, Accessed: May 28, 2024. [Online]. Available: https://engineering.stanford.edu/magazine/article/one-kind-wind-tunnel-birds-paves-way-better-drones.

[2] Towing Tank | Ocean and Naval Architectural Engineering, Memorial University of Newfoundland. Accessed: May 28, 2024. [Online]. Available: https://www.mun.ca/engineering/ona/research/research-facilities/towing-tank/.

[3] Gad-el-Hak M. (Sep. 1987). The water towing tank as an experimental facility. Exp. Fluids.

[4] Rodríguez-Sevillano Á.A., Casati-Calzada M.J., Bardera-Mora R., Feliz-Huidobro A., Calle-González C., Fernández-Antón J. (Dec. 2022). Flow study on the anemometers of the perseverance based on towing tank visualization. Appl. Mech..

[5] Yu C.-M., Lin Y.-H. (Jan. 2020). Experimental analysis of a visual-recognition control for an autonomous underwater vehicle in a towing tank. Appl. Sci..

[6] Morooka C., Tsukada R. (Oct. 2013). Experiments with a steel catenary riser model in a towing tank. Appl. Ocean Res..

[7] Descartin J.A. (Aug. 2022). Towing tank and wave basin facility: first of its kind in the Philippines. Am. J. Model. Optim..

[8] Rahaman M., Akimoto H., Orihara H., Zakaria N., Shabnam S. (Jul. 2014). Prospects of virtual or computational towing tank facility for the shipbuilding industry of Bangladesh. J. Nav. Archit. Mar. Eng..

[9] MIT Towing Tank | Facilities. Accessed: May 28, 2024. [Online]. Available: https://web.mit.edu/towtank/www/facilities.html.

